# *Loxosceles gaucho* Venom-Induced Acute Kidney Injury – In Vivo and In Vitro Studies

**DOI:** 10.1371/journal.pntd.0001182

**Published:** 2011-05-31

**Authors:** Rui V. Lucato, Regina C. R. M. Abdulkader, Katia C. Barbaro, Glória E. Mendes, Isac Castro, Maria A. S. F. Baptista, Patrícia M. Cury, Denise M. C. Malheiros, Nestor Schor, Luis Yu, Emmanuel A. Burdmann

**Affiliations:** 1 Division of Nephrology, São José do Rio Preto Medical School, São José do Rio Preto, Brazil; 2 Division of Nephrology, São Paulo Federal University, São Paulo, Brazil; 3 Division of Nephrology, University of São Paulo Medical School, São Paulo, Brazil; 4 Laboratory of Immunopathology, Butantan Institute, São Paulo, Brazil; 5 Department of Pathology, São José do Rio Preto Medical School, São José do Rio Preto, Brazil; Liverpool School of Tropical Medicine, United Kingdom

## Abstract

**Background:**

Accidents caused by *Loxosceles* spider may cause severe systemic reactions, including acute kidney injury (AKI). There are few experimental studies assessing *Loxosceles* venom effects on kidney function in vivo.

**Methodology/Principal Findings:**

In order to test *Loxosceles gaucho* venom (LV) nephrotoxicity and to assess some of the possible mechanisms of renal injury, rats were studied up to 60 minutes after LV 0.24 mg/kg or saline IV injection (control). LV caused a sharp and significant drop in glomerular filtration rate, renal blood flow and urinary output and increased renal vascular resistance, without changing blood pressure. Venom infusion increased significantly serum creatine kinase and aspartate aminotransferase. In the LV group renal histology analysis found acute epithelial tubular cells degenerative changes, presence of cell debris and detached epithelial cells in tubular lumen without glomerular or vascular changes. Immunohistochemistry disclosed renal deposition of myoglobin and hemoglobin. LV did not cause injury to a suspension of fresh proximal tubules isolated from rats.

**Conclusions/Significance:**

*Loxosceles gaucho* venom injection caused early AKI, which occurred without blood pressure variation. Changes in glomerular function occurred likely due to renal vasoconstriction and rhabdomyolysis. Direct nephrotoxicity could not be demonstrated in vitro. The development of a consistent model of *Loxosceles* venom-induced AKI and a better understanding of the mechanisms involved in the renal injury may allow more efficient ways to prevent or attenuate the systemic injury after *Loxosceles* bite.

## Author Summary

*Loxosceles* (recluse or brown spider) is the most important spider genus causing human envenomation. In Brazil *Loxosceles* spiders were responsible for approximately 7,000 cases of spider envenomation per year. The brown spider accidents may cause late cutaneous necrosis at the bite site, intravascular hemolysis, rhabdomyolysis, coagulation system changes and acute kidney injury (AKI). Even patients with mild cutaneous lesion may develop severe hemolysis and AKI, which is the main cause of death after these accidents. The mechanisms causing kidney injury are poorly understood. In this manuscript we described a consistent rodent model of *Loxosceles gaucho* venom-induced AKI and studied some of the possible mechanisms of the renal lesion. The results of this research showed that kidney injury may occur independently of the cutaneous lesion and without changes in the systemic blood pressure. Kidney dysfunction occurred likely due to intra-renal vasoconstriction and rhabdomyolysis, although a direct toxic effect of the venom on the proximal tubules cannot be ruled out.

## Introduction

*Loxosceles* spiders can be found worldwide in temperate and tropical regions but their distribution is heavily concentrated in the Western Hemisphere [1], [2]. In fact, in South America, loxoscelism is considered the most important form of araneism due to its high incidence and morbidity [1]–[3]. In Brazil, *Loxosceles* spiders were responsible for approximately 7,000 cases of spider envenomation reported to the Brazilian Ministry of Health in 2006 [Unpublished data. SINAN-Animais Peçonhentos/SVS/MS. http://dtr2004.saude.gov.br/sinanweb/tabnet/dh?sinan/animaisp/bases/animaisbr.def].

*Loxosceles* venom is a complex mixture of several proteins including alkaline phosphatase, hyaluronidase, 5-ribonucleotidase phosphohydrolase, sphingomyelinase D, several proteases, esterase and ATPase. Sphingomyelinase D is considered the most toxic fraction of the venom, playing a key role in its local and systemic action [1]–[4]. It causes neutrophil migration, complement system activation, cytokine and chemokine release and platelet aggregation [5].

*Loxosceles* spiders are not aggressive and the bites usually occur when they are pressed against the body, mainly while the victim is sleeping or dressing. The accident may cause mild cutaneous inflammatory reaction or a local injury characterized by pain, edema and livedo, developing later to dermonecrosis with gravitational spreading [1]–[3]. In up to 13% of the cases [1], loxoscelism can cause a systemic injury, known as viscerocutaneous loxoscelism (VCL). This form occurs predominantly in children [6], and patients may develop acute kidney injury (AKI), which is considered the main cause of death after loxoscelism envenomation [5], [7]. VCL is characterized by fever, malaise, weakness, nausea and vomiting, hemolysis, hematuria, jaundice, thrombocytopenia and disseminated intravascular coagulation. This severe multisystemic clinical picture can occur as early as 24 hours after the bite [1]–[3], [7].

AKI has been described in VCL as single case reports [7]–[11] or as relatively small series of cases [12]–[14]. Data on AKI after VCL are not consistent, even in the same country. Several factors might account for this, including the spider species and the patient age. In Brazil, 49% of AKI, 45.7% of oliguria and 8.6% of anuria were found among 35 VCL cases [6]. On the other hand, among 359 cases treated in Butantan Institute, Brazil, 4% developed VCL and none presented AKI [15]. In Chile, plasma creatinine was assessed in 26 of 34 VCL cases and was elevated in all, with values ranging from 4.4 to 6.0 mg/dL [16]. In the USA, AKI was found in 1 of 6 children hospitalized due to VCL [17]. These differences in AKI frequency can be due to the distribution of different *Loxosceles* species through the North and South Americas [5]. In São Paulo the commonest specie is the *Loxosceles gaucho* but in other regions of Brazil the *Loxosceles intermedia* is the most prevalent. The mechanisms for *Loxosceles* venom-induced AKI are still elusive and renal injury has been attributed to hemolysis, rhabdomyolysis, shock and direct venom nephrotoxicity [1], [2], [5], [11], [14], [17]–[19].

Few experimental studies have focused on the action of the *Loxosceles* venom in the kidney, and none have performed a detailed study on renal function and hemodynamics. The aim of the present study was to assess the nephrotoxicity of *Loxosceles gaucho* venom in rats and study some of the mechanisms possibly involved in the genesis of the renal injury.

## Materials and Methods

### Ethics Statement

Experiments were done according to the Brazilian law of protection of animals. The study was approved by the Animal Experimentation Ethics Committee (CEEA), FAMERP.

### Venom

Specimens of *L. gaucho* were collected in São Paulo State. The spiders were kept in quarantine for one week without food before venom collection, and venoms were obtained as previously described [20]. A pool of venom collected from approximately 1,000 *L. gaucho* spiders was used. The protein content of venom pool was determined using bicinchoninic acid [21].

### In vivo experiments

#### Animals

Adult male Wistar rats weighing 150 to 270 g were housed in a temperature- and light-controlled environment. They received standard diet and were allowed free access to tap water.

#### Venom infusion

*Loxosceles gaucho* venom (LV) was diluted in saline and infused (240 µg/kg) in the jugular vein at the rate of 0.06 mL/min (infusion pump, model 975, Harvard, USA). Control animals received the same volume of saline. The LV dose used was selected after pilot experiments beginning with 0.15 mg/kg that was the LD_50_ for mice after subcutaneous injection [22].

#### Glomerular filtration rate (GFR)

After anesthesia with intraperitoneal thiopental (50 mg/kg) tracheostomy was performed and a polyethylene tube (PE-50) was placed in a carotid artery. Mean carotid arterial blood pressure (BP) was continuously measured with an electronic transducer (P23 Db, Statham Instruments Division, Could Inc., Hato Rey, USA) connected to a digital polygraph (Blood Pressure Display Unit, Stoelting, USA) throughout the experiments. PE-50 tubes were also placed in both jugular veins and a polyethylene tube (PE-90) was placed and sutured in the bladder. Animals were maintained in a stable temperature (36.5±1°C) by a thermostatically controlled warming table (Braile Biomédica, São José do Rio Preto, Brazil). After surgery, a dose of 1 mL of inulin solution (300 mg of inulin in 12 mL of saline, Sigma Chemical Co., St. Louis, MO, USA) was given, followed by an infusion of the same solution at a rate of 0.06 mL/min (Harvard infusion pump model 975, USA). After a 60 min equilibration time, LV (venom, n = 8) or saline (control, n = 8) were infused as described above. After a 30 min equilibration time, urine was collected over two periods of 20 min each. A blood sample (0.3 mL) was drawn at the midpoint of each urine collection and replaced with an equal volume of saline. Inulin clearance values, expressed as mL/min/100 g body wt, represent the means of the two clearance periods. Diuresis (µL/min) was assessed by the weight difference in the collection vials before and after urine collection.

#### Renal blood flow (RBF)

Anesthesia, tracheostomy and carotid and jugular surgical preparation were performed as previously described. After that, a ventral midline incision was made, exposing the left renal artery, which was dissected and a suitable probe (R series, 1.5 mm, Transonic Systems, Ithaca, NY, USA) was placed around it. At this point LV (venom, n = 8) or saline (control, n = 8) were infused as already described, followed by maintenance infusion of saline at 0.06 mL/min (infusion pump, Harvard Apparatus, Holliston, MA, USA).

After a 60 min equilibration time, four ultrasonic RBF measurements were performed (T 106, Transonic Systems Inc, Ithaca, NY, USA) during 10 min observation period. RBF values represent the means of the four measurements. BP was continuously monitored as previously described. Renal vascular resistance (RVR) was calculated by the usual formula (BP/RBF).

#### Hemolysis and rhabdomyolysis evaluation

Anesthesia, surgical preparation and venom (venom, n = 8) or saline (control, n = 8) infusion were carried out as previously described. After 60 minutes of equilibration period blood was collected for measurement of hematocrit (Hct), creatine kinase (CK), serum aspartate aminotransferase (AST), serum alanine aminotransferase (ALT) and lactic dehydrogenase (LDH).

### Renal histology

The left kidney was collected at the end of GFR measurements in 6 rats injected with LV and 6 rats injected with saline. Renal tissue was fixed in 4% buffered formalin and embedded in paraffin. Horizontal sections 3 to 4 µm thick were stained with periodic acid-Schiff's reagent (PAS), hematoxylin-eosin, Masson trichrome and periodic acid methenamine silver. The tissue was evaluated by light microscopy by two observers masked to the treatment. Tubular, vascular and glomerular changes were evaluated according a 0 to 3 semiquantitative score. In the vessels and glomeruli, signs of endothelial injury (fibrin thrombi in glomerular capillary lumina, disruption or reduplication of the glomerular capillary basement membrane, swollen endothelial cells and fibrinoid necrosis) were extensively searched.

### Immunohistochemistry

Sections from representative formalin-fixed paraffin embedded samples were stained with monoclonal antibody against hemoglobin and myoglobin, with amplification by streptavidin-peroxidase method. Briefly, after deparaffinization in xylene and rehydration in graded ethanol, antigen epitope retrieval was performed using 10 mM citric acid solution, pH 6.0 in a pressure cooker. Endogenous peroxidase activity was blocked with 6% hydrogen peroxide for 20 min.

Primary mouse Polyclonal Rabbit Anti-human Hemoglobin antibody (code # A0118, DakoCytomation, USA), diluted 1∶1000, and Sheep Anti-human Myoglobin (code # PH 213, The Binding Site, USA), diluted 1∶50, were incubated for 30 min at 37°C followed by overnight incubation at 4°C, and then by addition of biotinylated anti-mouse secondary antibody and streptavidin-horseradish peroxidase (LSAB+, code # k0690, Dako, Carpinteria, CA, USA).

Color of reaction product was developed by 3,3′-diaminobenzidine and H_2_O_2_ and counterstaining was performed with Harris hematoxylin. The primary antibody was omitted for negative controls and endothelial cells of tonsil were used as positive control. The immuno-expression of hemoglobin and myoglobin was evaluated in a semi-quantitative approach. Samples with no evidence of staining or those with evidence of only focally positive cells (<1%) were recorded as negative.

### In vitro experiments

#### Proximal tubule injury

Isolation of proximal tubules (PT): male Wistar rats were anesthetized with sodium thiopental (40 mg/kg i.p.). PT were isolated by collagenase digestion and Percoll gradient as previously described [23].

PT toxicity protocol: LV venom concentrations of 3.17 and 6.34 µg/mL were added at baseline to oxygenated PT suspensions and kept for 60 min.

Cell injury: Cell injury was assessed by LDH release. LDH was measured after removal of 1 mL sample from tubules suspension (venom and time control groups) at baseline and after 60 min. LDH release was calculated by dividing supernate LDH by total LDH (supernate + pellet) and expressed as a percentage (n = 5).

### Analytical methods

Inulin was determined by chemical anthrone method. CK, AST, ALT and LDH were assessed by colorimetry (Hitachi auto-analyzer model 917, Japan). Hct was assessed by a microhematocrit method.

### Statistical analysis

Results are presented as mean ± standard error of mean (SEM). Comparisons were done by two-tail unpaired Student's t-test or one-way ANOVA, as appropriate. *P* values<0.05 were considered significant.

## Results

### Renal function and hemodynamics

*Loxosceles gaucho* venom caused an early and significant decrease in GFR and urinary volume. In the same way, venom caused a sharp and significant decrease in RBF and a significant RVR increase. These changes occurred without significant variation in BP. In the control group saline infusion did not change GFR, RBF and RVR, urinary volume or blood pressure (see table1).

**Table 1:** Renal and systemic parameters after administration of *Loxosceles* venom (venom) or saline (control).

	Control (n = 8)	Venom (n = 8)	P
GFR (mL/min/100 g)	0.92±0.06^a^	0.30±0.04	p<0.0001
RBF (mL/min)	4.6±0.3	1.9±0.2	p<0.0001
RVR (mmHg/mL/min)*	21.4±2.4	58.6±15.0	p<0.005
BP (mmHg)	132±5	126±4	NS
Diuresis (µL/min)	9.4±0.3	5.2±0.6	p<0.0001
CK (IU/L)	598±82	1338±220	p<0.005
LDH (IU/L)	91±14	323±140	NS
ALT (IU/L)	57±4	226±128	NS
AST (IU/L)	121±4	283±66	p<0.005
Hematocrit (%)	48±1	47±2	NS

a Mean ± SE; GFR: glomerular filtration rate; RBF: renal blood flow; RVR: renal vascular resistance; BP: blood pressure; CK: creatine kinase; LDH: lactic dehydrogenase; ALT: alanine amintransferase; AST: aspartate aminotransferase;

*n = 6 for RVR; NS: not statistically significant.

### Direct tubular toxicity

*Loxosceles gaucho* venom did not cause cellular injury to PT in both concentrations utilized. After 60 min LDH release was 21.2±0.4% in control, 22.0±1.0% in LV 3.17 µg/mL and 22.4±0.5% in LV 6.34 µg/mL.

### Enzymes and hematocrit

*Loxosceles gaucho* venom induced a statistically significant increase in serum CK and AST as compared to control group. LDH and ALT values also increased in the venom group, but the difference was not statistically significant when compared to saline-infused rats. Hematocrit values were similar in venom and control groups (see table 1).

### Histology

In the LV group, rats showed flattened epithelium (score 1.3±0.2), tubule dilatation (score 1.8±0.3), presence of cell debris in tubular lumen (score 1.25±0.25) presence of detached epithelial cells in tubular lumen (score 1.2±0.2) and acute epithelial degenerative changes (score 1.6±0.3). In the control group, animals showed normal renal histology. Endothelial injury was not found in any animal. All glomerular capillaries showed patent lumina and preserved endothelial cells.

### Immunohistochemistry

The control group showed negative myoglobin immunostaining in all rats. In the LV group, three animals presented focal myoglobin deposition (1+), one animal disclosed focal and interstitial myoglobin staining (1+), one animal showed interstitial myoglobin deposition (2+) and one animal did not present myoglobin at renal tissue.

In the control group two animals presented focal hemoglobin staining (1+) and four animals were negative for hemoglobin. In the LV group, four animals showed focal hemoglobin staining (1+), one animal showed (3+) of hemoglobin deposition, and one animal did not present hemoglobin at renal tissue.

**Figure 1:**
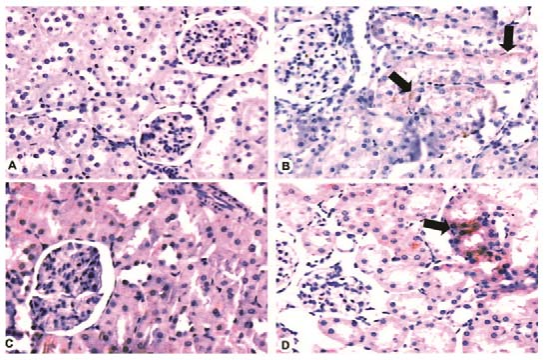
Immunohistochemistry for myoglobin and hemoglobin in the renal tissue of control and venom-injected rats. A. Negative staining for myoglobin (200 X). B. Positive tubular cytoplasm staining for myoglobin (black arrows). Note tubular necrosis in the same area (200 X). C. Negative staining for hemoglobin (200 X). D. Positive tubular cytoplasm staining (black arrows) for hemoglobin (200 X).

These data are represented at Figure 1.

## Discussion

An important and novel result from this study is the finding that *Loxosceles gaucho* venom may produce renal damage and rhabdomyolysis independently from the dermonecrotic injury or to blood pressure changes. The factors most likely contributing for the observed renal injury were renal vasoconstriction and myoglobinuria. In a consistent way with the available clinical results [14], [16], [24], [25] and to data originated from experimental studies in mice [18], [19], [26], renal histology disclosed acute tubular necrosis.

There was an early and intense RBF decrease and RVR increase in venom-infused animals. *Loxosceles* venom may cause intravascular clotting and consequently tissue ischemia [27], [28], but this phenomenon was not observed in the present study. Other mechanisms possibly related to LV-induced decrease in RBF are the venom toxicity to endothelial cells [29], its property to degrade extracellular matrix molecules acting against basement membrane structures [30] and its vasoconstrictive activity [5]. Contrasting with the findings of an experimental model that utilized *Loxosceles intermedia* venom [18], no signs of endothelial injury were found in the present study, in which *Loxosceles gaucho* venom was used. These differences might explain the variation in lethality of the venom of diverse *Loxosceles* species. In fact, *Loxosceles intermedia* venom is more lethal than *Loxosceles gaucho* venom [31].

Rhabdomyolysis has been sporadically related after human loxoscelism [7], [14], [17]. Although only low myotoxicity activity was reported in experimental studies with LV [31], [32], the venom clearly caused rhabdomyolysis in the current experiment, as evidenced by significant CK increase and deposition of myoglobin in renal tissue. It is noteworthy that rhabdomyolysis was caused by a systemic venom effect, totally independent from local injury, which actually did not exist in the present model. Rhabdomyolysis is a well known cause of AKI, causing direct injury to tubule cells and inducing or enhancing vasoconstriction [33].

*Loxosceles* envenomation has been associated to hemolysis in humans [5], [7], [34]. Although there was no hematocrit decrease in the venom-infused rats, renal tissue stained positively for hemoglobin after venom infusion and LDH was three times higher in the venom as compared to the control group. Even considering that this difference was not statistically different, it is possible that some degree of hemolysis had occurred, contributing for the renal injury genesis.

The early development of renal dysfunction, the low molecular weight and the cationic charge of the venom components, facilitating its renal excretion, suggest that LV might have a potential direct toxic effect on tubule cells. In fact, Luciano et al. [18] showed *L. intermedia* venom binding at glomerular and tubule cells basement membranes of mice with venom-induced AKI. Considering that this venom is capable to damage the basement membrane, a direct nephrotoxicity effect may have contributed to AKI development in these mice. Chaim et al. [19] demonstrated the deposit and binding of the dermonecrotic fraction of *L. intermedia* venom to renal intrinsic structures of mice with LV-induced renal injury. When the venom was added to a MDCK cell culture, it deposited in the cells surface and caused cells structural changes, impaired spreading and cells adhesion and altered cell viability. The current results are discordant with this previous paper, since *Loxosceles* venom did not cause direct toxicity in proximal tubules suspension. However, there are some possible explanations for this difference. While Chaim et al. [19], used cultured MDCK cells, we used fresh prepared proximal tubules suspension from rats. The spider species utilized were different, *L. gaucho* in the present experiment versus *L. intermedia* in the other cited studies [18], [19]. Finally, it is possible that the venom needs plasma components, such as complement, not present in our experimental preparation, in order to cause proximal tubule cell toxicity.

There is just another study assessing the effects of *Loxosceles* venom in vivo, which was performed in mice [18]. The authors didńt measure glomerular filtration rate, systemic and renal hemodynamic were not assessed, neither muscle enzymes measured. They found blood urea elevation four hours after intraperitoneal injection of crude *L. intermedia* venom. There was no hemolysis and renal histology analysis disclosed glomerular collapse and loss of vascular integrity and tubule cell injury. In a subsequent study, the same group demonstrated that the intraperitoneal injection of the dermonecrotic fraction of the venom in mice caused nephrotoxicity similar to that seen with the crude venom [19].

In summary, intravenous injection of *Loxosceles gaucho* venom induced a striking acute kidney injury, without dermonecrotic lesion or variations in systemic blood pressure. The observed renal changes were associated to impaired renal blood flow and systemic rhabdomyolysis. Although a direct venom effect on isolated proximal tubules was not demonstrated, venom direct nephrotoxicity cannot be totally ruled out.
